# High-dimensional mediation analysis in survival models

**DOI:** 10.1371/journal.pcbi.1007768

**Published:** 2020-04-17

**Authors:** Chengwen Luo, Botao Fa, Yuting Yan, Yang Wang, Yiwang Zhou, Yue Zhang, Zhangsheng Yu

**Affiliations:** 1 Department of Bioinformatics and Biostatistics, School of Life Sciences and Biotechnology, Shanghai Jiao Tong University, Shanghai, China; 2 SJTU-Yale Joint Center for Biostatistics, Shanghai Jiao Tong University, Shanghai, China; 3 Department of Biostatistics, University of Michigan, Ann Arbor, Michigan, United States of America; Institute for Disease Modeling, UNITED STATES

## Abstract

Mediation analysis with high-dimensional DNA methylation markers is important in identifying epigenetic pathways between environmental exposures and health outcomes. There have been some methodology developments of mediation analysis with high-dimensional mediators. However, high-dimensional mediation analysis methods for time-to-event outcome data are still yet to be developed. To address these challenges, we propose a new high-dimensional mediation analysis procedure for survival models by incorporating sure independent screening and minimax concave penalty techniques for variable selection, with the Sobel and the joint method for significance test of indirect effect. The simulation studies show good performance in identifying correct biomarkers, false discovery rate control, and minimum estimation bias of the proposed procedure. We also apply this approach to study the causal pathway from smoking to overall survival among lung cancer patients potentially mediated by 365,307 DNA methylations in the TCGA lung cancer cohort. Mediation analysis using a Cox proportional hazards model estimates that patients who have serious smoking history increase the risk of lung cancer through methylation markers including cg21926276, cg27042065, and cg26387355 with significant hazard ratios of 1.2497(95%CI: 1.1121, 1.4045), 1.0920(95%CI: 1.0170, 1.1726), and 1.1489(95%CI: 1.0518, 1.2550), respectively. The three methylation sites locate in the three genes which have been showed to be associated with lung cancer event or overall survival. However, the three CpG sites (cg21926276, cg27042065 and cg26387355) have not been reported, which are newly identified as the potential novel epigenetic markers linking smoking and survival of lung cancer patients. Collectively, the proposed high-dimensional mediation analysis procedure has good performance in mediator selection and indirect effect estimation.

## Author summary

In this research, we established an efficient procedure for high-dimensional mediation analysis with time-to-event data to select DNA methylation markers and estimate the mediation effects. To evaluate the performance of the proposed procedure, we conducted extensive simulation studies and analyzed a lung cancer data set using our method. We demonstrated the validity and utility of our method under a variety of scenarios.

## Introduction

Mediation analysis based on counterfactuals has been widely used in understanding the causal pathways from an exposure to an outcome. The idea of mediation approach was firstly applied in psychology research [1–3], and gradually extended to other fields including epidemiology, biomedical, and clinical studies. Through mediation models, the relationships among exposure, mediator, and outcome can be characterized. Specially, the graphical model of causal mediation models can be illustrated by using a directed acyclic graph (DAG) [4]. Moreover, through mediating effect analysis, the total effect of an exposure on the outcome is decomposed into two parts. One is the natural direct effect, which is the effect of an exposure directly on the outcome that is not through mediators. Another part is the natural indirect effect, which describes the effect of an exposure on the outcome through the mediators.

Extensive works have been done in mediation analysis during the past decades, particularly in the area of causal inference [4–9]. Besides, mediation analysis has been generalized from continuous outcomes to binary outcomes [10–12], even to the time-to-event outcome [13,14], since many epigenetic questions involve addressing censored survival data. Recent years have seen huge progress in the extensive of mediation methods to survival models. For instance, built upon the framework of causal inference, the methodology of mediation analysis has a pervasive application with Aalen’s additive hazards model, Cox proportional hazards model and accelerated failure time model [15,16].

To date most methodology in mediation analysis has been concentrated on the context of a single mediator, with only few attention relating to multiple mediators [17,18], especially in survival data [19]. However, scare attention has been received for the development of approaches to deal with mediation with high-dimensional mediators. As rapid advances in technology have generated large amount of data from genome or genetic researches, there is a broad application of mediation analysis for high-dimensional data [20–29]. For example, Zhang et al. (2016) considered estimating and testing mediation effects for high-dimensional epigenetic data and showed that DNA methylation is mediated between smoking and lung functions [20]. Nevertheless, methods for high-dimensional mediation analysis with survival outcomes are still yet to be developed. Such an extension is the aim of this work.

As a motivating example, smoking has side effects on human health, especially for lung cancer which is the leading cause of cancer mortality worldwide and creates an enormous public health burden [30]. When individual level phenotype and genotype data are available, numerous researches have indicated that mutation in CpG sites are related to tobacco smoking [31–35]. It is of great scientific interest to identify which methylation markers are acting as mediators between smoking and lung cancer patient’s survival, as this is essential for finding the disease diagnosis markers and the treatment target in precision medicine. In practice, both Illumina Infinium HumanMethylation27 and HumanMethylation450 are widely used platform which allow to measure DNA methylation levels of roughly 27k and 450k respectively. High-dimensional methylation data are generated in both platforms [36]. Hence, it is of great importance to identify the significant mediators among the huge number of potential candidates in the survival models. Previous studies utilized continuous or binary outcome when selecting the high-dimensional mediators [20]. However, when in the context of survival analysis, such method will lose efficiency as it ignores the time-to-event information.

In this article, we aim to study the selection of mediators (DNA methylations) between the smoking exposure and the overall survival in lung cancer patients. We propose a procedure to select, estimate and test mediation effects in survival models with high-dimensional epigenetic information. The main idea is as follows. Firstly, we reduce the dimension of potential mediators from ultra high-dimensional to moderate (i.e., one that is less than the sample size) using sure independence screening (SIS) method [37]. Secondly, we conduct variable selection via Cox proportional hazards model with the minimax concave penalty (MCP) [38]. Finally, we decompose the total effect and carry out the Sobel and joint significance test for mediation effect. This is the first proposed procedure for mediator identification in the survival models, to the best of our knowledge.

The rest of the paper proceeds as follows. In the next part, we provide simulation studies to evaluate the performance of our proposed procedure and a real data application to analyze the mediation effects of high-dimensional DNA methylation markers on the causal effect of smoking on lung cancer in a epigenome-wide study. Then, we conclude the paper through discussing limitations and other feasibilities. Finally, we introduce models, assumptions and develop the proposed procedure.

## Results

### Simulation studies

This section is devoted to a series of simulation studies which evaluate the performance of the proposed method with time-to-event outcome. We demonstrate the performance of the proposed procedure through mediator selection and indirect effect estimation.

#### Simulation design

We generate death time from the exponential model as follows, *λ*_*i*_(*t*|*X*_*i*_,*M*_*i*_) = 0.5 exp{*γX*_*i*_+*θ*_1_*Z*_1*i*_+*θ*_2_*Z2*_*i*_+*β*_1_*M*_*1i*_+⋯*β*_*p*_*M*_*pi*_} for subject *i* = 1,2,⋯,*n*, where the exposure *X* is generated from binary distribution as *B*(1,0.6) with *γ* = 0.5; the covariate *Z*_1_ is generated from binary distribution as *B*(1,0.3), which we used to represent gender; the covariate *Z*_2_ is generated from *U*(0,1), which we used to represent age; *θ*_1_ = 0.3 and *θ*_2_ = −0.2 are represented for the effects of *Z*_1_ and *Z*_2_ on the outcome. The mediators are generated from *M*_*ik*_ = *c*_*k*_+*α*_*k*_*X*_*i*_+*ϑ*_1_*Z*_1*i*_+*ϑ*_2_*Z*_2*i*_+*e*_*ik*_,*k* = 1,2,⋯,*p*, where *c*_*k*_ is chosen as a random number from *U*(0,1); *e*_*ik*_ is generated from *N*(0,1); and (*ϑ*_1_,*ϑ*_2_) = (0.3,0.2). The number of mediators is set as *p* = 10,000. The first eight elements of *α* are (0.5,0.45,0.5,0.4,0.45,0.45,0,0), and the first eight elements of *β* are (0.55,0.6,0.65,0.7,0,0,0.5,0.5). The rest of elements of *α* and *β* are set as 0. The censoring time is generated from *U*(0,*c*_0_) distribution with constant *c*_0_ chosen so that we can control the percentage of censored subjects. We perform the simulation under three levels of censoring percentages of 15%, 25%, and 35% proximately and three sample sizes *n* = 300,500,1000. For each scenario, we generate 500 replicates.

#### Simulation results

We perform the analysis using the proposed procedure with time-to-event outcome and the simulation results are summarized in Tables 1 and 2. We present true positive rate (TPR, percentage of nonzero mediators correctly selected), the number of false positive (FP, the number of zero mediators incorrectly selected), and false discovery proportion (FDP, percentage of incorrect selection among all selected).

**Table 1:** Accuracy of mediator selection (p = 10000, with 500 replications).

Censoring	Sample size	Sobel test			Joint test		
		TPR	FP	FDP	TPR	FP	FDP
15%	300	0.7860	0.0080	0.0019	0.8400	0.2360	0.0519
	500	0.9865	0.0060	0.0012	0.9900	0.0340	0.0069
	1000	1	0.0220	0.0044	1	0.0360	0.0072
25%	300	0.7650	0.0100	0.0025	0.8355	0.2460	0.0581
	500	0.9840	0.0060	0.0012	0.9880	0.0360	0.0074
	1000	1	0.0200	0.0040	1	0.0280	0.0056
35%	300	0.7435	0.0080	0.0019	0.8270	0.2480	0.0584
	500	0.9850	0.0080	0.0016	0.9880	0.0500	0.0099
	1000	1	0.0220	0.0044	1	0.0300	0.0060

*TPR: the average value of true positive rates; FP: the average number of false positive; FDP: false discovery proportion (= V/R, where V is the number of false discoveries, R is the number of total discoveries); TPR, FP and FDP are the average value over 500 times.

**Table 2:** Estimation of log hazard indirect effects: *α*_*k*_*β*_*k*_.

(α_k_,β_k_) = α_k_β_k_	Estimation	Cen = 15%			Cen = 25%			Cen = 35%		
		n = 300	n = 500	n = 1000	n = 300	n = 500	n = 1000	n = 300	n = 500	n = 1000
(0.5,0.55) = 0.275	Est. CP Emp. SE Est. SE	0.2952 0.9238 0.0855 0.0794	0.2794 0.9220 0.0642 0.0584	0.2753 0.9360 0.0425 0.0405	0.2954 0.9173 0.0874 0.0805	0.2794 0.9400 0.0658 0.0590	0.2758 0.9320 0.0430 0.0410	0.2956 0.9004 0.0935 0.0815	0.2801 0.9420 0.0670 0.0600	0.2764 0.9360 0.0435 0.0416
(0.45,0.6) = 0.27	Est. CP Emp. SE Est. SE	0.2916 0.9300 0.0892 0.0825	0.2806 0.9480 0.0616 0.0622	0.2716 0.9600 0.0417 0.0428	0.2930 0.9120 0.0954 0.0837	0.2821 0.9520 0.0636 0.0630	0.2717 0.9680 0.0419 0.0432	0.2930 0.9151 0.0981 0.0846	0.2827 0.9500 0.0647 0.0638	0.2719 0.9600 0.0424 0.0436
(0.5,0.65) = 0.325	Est. CP Emp. SE Est. SE	0.3443 0.9358 0.0936 0.0902	0.3343 0.9380 0.0701 0.0677	0.3300 0.9600 0.0459 0.0467	0.3445 0.9196 0.0970 0.0912	0.3341 0.9380 0.0698 0.0683	0.3305 0.9480 0.0467 0.0472	0.3436 0.9295 0.0973 0.0921	0.3347 0.9480 0.0704 0.0692	0.3311 0.9520 0.0473 0.0477
(0.4,0.7) = 0.28	Est. CP Emp. SE Est. SE	0.2975 0.9440 0.0959 0.0936	0.2889 0.9780 0.0641 0.0699	0.2813 0.9420 0.0479 0.0483	0.2978 0.9400 0.0990 0.0942	0.2897 0.9720 0.0654 0.0705	0.2814 0.9460 0.0483 0.0486	0.2982 0.9320 0.0988 0.0952	0.2896 0.9740 0.0664 0.0710	0.2815 0.9380 0.0485 0.0489
(0.45,0) = 0	Est. CP	0.0078 0.5714	- -	- -	0.0167 0.3157	0.0514 0	- -	0.0098 0.5384	0.0143 0	- -
(0.45,0) = 0	Est. CP	0.0327 0.1538	-0.0478 0	- -	0.0393 0.2142	0.0860 0	- -	0.0128 0.4736	-0.0485 0.5000	- -
(0,0.5) = 0	Est. CP	-0.0043 0.9621	0.0024 0.9620	0.0009 0.9520	-0.0051 0.9620	0.0024 0.9674	0.0009 0.9520	-0.0043 0.9631	0.0027 0.9708	0.0009 0.9520
(0,0.5) = 0	Est. CP	-0.0013 0.9656	0.0011 0.9440	0.0035 0.9540	-0.0013 0.9661	0.0012 0.9433	0.0035 0.9540	-0.0006 0.9629	0.0008 0.9445	0.0035 0.9540
(0,0) = 0	Est. CP	0.0382 1	- -	- -	0.0930 1	- -	- -	0.0621 1	- -	- -
(0,0) = 0	Est. CP	-0.0027 1	- -	- -	-0.0136 1	- -	- -	-0.0737 1	- -	- -

*Est.: the mean of estimators; CP: coverage probability, the proportion of the replicates that the 95% confidence interval covers the true value of estimate; Emp. SE: empirical standard error calculated as the sample standard deviation of the estimates over all replicates; Est. SE: the average of the standard errors over all replicates;—means the not available value.

We first assess the accuracy of variable selection with our proposed procedure. Table 1 shows the selection results of the proposed procedure. The TPRs are high among all the censoring and sample size settings with the lowest rate of 0.7435 at the high censoring rate setting (35%). Among all the 9,996 zero-effect mediators, the highest FP is 0.2480. The false discovery proportion (FDP) among the selected mediator is lower than 0.0584 among all settings. As the sample size increases to 500 and 1000, the TPR increases to about 1. Compared with the performance of identifying significant markers with the Sobel test, the joint test has higher TPRs and also a slight higher FPs and FDPs.

To highlight the effectiveness of our approach, we compare our procedure with other methods. One is the one-step approach, i.e., using MCP-based regularization alone. Another is the naive approach, i.e., fitting the mediator model and Cox model for each mediator. Our method shows better performance than one-step method and the naive method (S1 Table & S2 Table). Besides, we also conduct other simulations. The proposed method with MCP-based regularization performs slightly better than the LASSO-based regularization (S3 Table). As the number of significant mediator decreases, there is a higher accuracy of mediator selection (S4 Table). Moreover, the TPR decreases as the censoring rate increases, especially with limited sample size and higher censoring rate (S5 Table). Additionally, we also consider the cases that mediators are dependent. The TPR increases with the increases of correlation among the mediators when the correlation is not very large, but decreases when the correlation is large due to the collinearities among the mediators (S6 Table). Overall, the performance of proposed selection procedure is good in terms of selecting significant biomarker and controlling both the FP and FDP.

Except for mediator selection, we also perform the mediation effect estimation. We evaluate the estimation of *α*_*k*_*β*_*k*_ and present the results in Table 2. In general, the bias is small. Both the empirical and estimated variance are close to each other and decrease as the sample size increases. Also, the coverage probability tends to be 0.95 as the sample size increases. Besides, the accuracy of effect estimation decreases with the increase of noise and the dimension of mediators (S7 Table & S8 Table).

In summary, the simulation studies show that the selection accuracy of the high-dimensional mediation model is high and the estimators of indirect effect through the nonzero mediators have minimal bias. To further demonstrate the efficacy of the approach, we apply the propose procedure to analyze a lung cancer data set.

### Data application

Lung cancer is one of the deadliest cancer worldwide [30]. It can be categorized as non-small-cell lung cancer (account for almost 85%) and small-cell lung cancer (15%) [30]. Among lung cancer patients, tobacco smoking is a common risk factors. Besides, many researches suggest that DNA methylation markers may be the potential promoters for lung cancer. For example, hypermethylation of CpG islands in the promoter regions of genes was demonstrated as a common phenomenon in lung cancer [39]. In addition, tobacco smoking was related with methylation [31]. How the smoking behaviors affect the cancer survival through the methylation is of great interest. We apply the proposed procedure to identify which methylation markers are the potential mediators between the tobacco smoking and the overall survival time.

We applied the proposed method to the TCGA (The Cancer Genome Atlas) lung cancer cohort study including lung squamous cell carcinoma and lung adenocarcinoma. There were 1299 lung cancer patients aged 33–90 years. 907 of them had DNA methylation profile measured using the Illumina Infinium HumanMethylation 450 platform. DNA methylation values were recorded for each array probe in each sample via BeadStudio software. A total of 365,307 probes were included in the analysis.

To identify the potential methylation mediators between the tobacco smoking and the overall survival, we applied the high-dimensional mediator model with smoking status assessed at their initial diagnosis (smoker/non-smoker) as the exposure variable, DNA methylation measured at the same time as the high-dimensional mediators, and the survival time as the outcome variable. The overall survival time was defined as the number of days from initial diagnosis to the death or the last follow-up date. The median survival time was 54.4 months. Subject with no survival time, exposure and other covariates were excluded; we got 754 patients with 305 deaths observed during the follow-up. Other covariates including age at initial diagnosis, gender, tumor stage and radiotherapy (yes/no) were adjusted.

Due to the fact that the relationships between methylation and the outcome are much stronger than those between exposure and methylation in the analysis data set, we add top *d* = 3*n*/log(*n*) CpGs using sure independence screening method based on the path from smoking to the methylation (S1 Text) in order to improve the probability to recognize significant mediators. Secondly, we run a variable selection on the CpGs screened in the first step. Finally, we carry out the significance test for the direct and indirect effects.

The analysis results are presented in Table 3. We identified 6 CpGs mediating the relationship between smoking and overall survival of lung cancer patients with Bonferroni’s adjusted p-value<0.05. Since smoking generally increases the risk of lung cancer and reduces overall survival of lung cancer patients with the total effect 1.3248 (95%CI: 1.022, 1.717), we focus on the three of these mediators with the log-hazard indirect effect *α*_*k*_*β*_*k*_>0 (smoking increases the mortality), where *k* denotes cg21926276, cg27042065 and cg26387355. All the three genes in which methylation sites locate are correlated with lung cancer or tumor growth in previous studies. For example, the gene H19 (cg21926276 locate) is related with both lung cancer and tumor growth, methylation of which has been thought as a sensitive marker of tobacco history[40,41]. The gene CDCA3 (cg27042065 located) is also associated with lung cancer and survival of cancer patients [42–44], and Song and Yang (2018) have reported that gene LOC338797 (cg26387355 located) is related with progression of tumor in lung cancer patients [45]. Besides confirming the previously reported genes, the three CpGs (cg21926276, cg27042065 and cg26387355) are also identified as novel markers for the survival of lung cancer patients. Besides, other methylation sites with negative log-hazard mediation effect have not been reported so far, and they may be the potential biomarkers to extend survival time for lung cancer patients. Take cg07690349 as an example, the gene MUC5B (cg07690349 locates) is one of the secreted mucins which are large O-glycosylated proteins that participate in the protection of underlying mucosae in normal adults [46].

**Table 3:** Summary of selected CpGs with estimators and *P*-values for significant mediators.

CpGs	Chromosome	Gene	$\hat{\alpha }\hat{\beta }$	*P*(Sobel)	*P*(Joint)
cg21926276	chr11	H19	0.2229	1.266e-03	1.662e-06
cg27042065	chr12	CDCA3	0.0880	1.071e-01	4.409e-02
cg26387355	chr12	LOC338797	0.1388	1.449e-02	1.558e-03
cg15292688	chr18	ZNF519	-0.2301	4.844e-02	1.084e-02
cg24200525	chr12	SBF1	-0.1127	3.018e-02	5.535e-03
cg07690349	chr11	MUC5B	-0.1403	1.217e-02	9.126e-04

*The CpGs are the DNA methylation sites. Chromosomes and Genes are where the CpGs locate. $\hat{\alpha }\hat{\beta }$ is the estimation of log-hazard indirect effect. *P*(Sobel) is the Sobel test p-values and *P*(Joint) is the joint test p-values, which are corrected by bonferroni’s method.

We are also interested in how the effect of the exposure is mediated through the DNA methylation markers. The path-specific effects of tobacco smoking on overall survival of lung cancer patients are listed in Table 4. Mediation analysis using Cox proportional hazards model discovers that the effect of having serious smoking history on increased risk of developing lung cancer is mediated through methylation markers including cg21926276, cg27042065, and cg26387355; the hazard ratio for each mediator is 1.2497(95%CI: 1.1121, 1.4045), 1.0920(95%CI: 1.0170, 1.1726), and 1.1489(95%CI: 1.0518, 1.2550), respectively. The direct effect is 1.4309(95%CI: 1.0806, 1.9074). Interventions can be explored on these markers to improve medical care for detection and treatment of lung cancer among smokers. Besides, we also use the one-step method and the naive approach for the lung cancer data, and they fail to identify any significant mediators.

**Table 4:** Path-specific effects (effect scale: hazard ratio) of tobacco smoking on overall survival of lung cancer patients (only CpGs with $\hat{\alpha }\hat{\beta }>0$ are included).

	Effect Estimate	95% Confidence Interval
X→Y(Direct effect)	1.4309	(1.0810, 1.9074)
X→cg21926276→Y	1.2497	(1.1121, 1.4045)
X→cg27042065→Y	1.0920	(1.0170, 1.1726)
X→cg26387355→Y	1.1489	(1.0518, 1.2550)
Total effect	1.3248	(1.0220, 1.7170)

*$e^{\left((\hat{\alpha })*(\hat{\beta })\right)}$ denotes the estimate effect.

Through the mediation analysis of DNA methylation for the survival time of the lung cancer patients, we found the three CpGs mediating the smoking and the mortality. Our findings not only were in line with previous studies which found that the gene that CpGs locate were important biomarkers for lung cancer [40], but also uncovered the mediation role of the markers connecting the smoking exposure and the survival time.

## Discussion

Identifying the right targets among large-scale potential epigenetic mediators is crucial in biomedical research. High-dimensional mediation analysis not only finds the potential interventional targets, but also connects the exposure and outcome through the identified targets. Finding the significant mediators can also help early detection of lung cancer and hence improve overall survival. In this article, we proposed a high-dimensional mediation survival model utilizing the time-to-event outcome in place of binary outcome to enhance accuracy of variable selection and minimize the estimation bias. Our approach involves sure independence screening, MCP penalized variable selection, as well as the Sobel and joint significance test and effect decomposition.

In this research, we established a facile and efficient procedure for high-dimensional mediation analysis with time-to-event data to select DNA methylation and estimate the effects of exposure and outcome mediated by the mediators. The proposed procedure has good performance in mediator selection and indirect effect estimation which has been showed in the simulation studies and real data analysis. We demonstrate the validity and utility of the proposed method through simulation studies and a TCGA lung cancer data example. The proposed method has high proportion in true positives and shows a well performance in controlling false positives and false discoveries. The proposed method can be widely used in biomedical data analysis, especially involving high-dimensional mediators.

For high-dimensional mediator analysis, many questions are still yet to be answered and of interest to future studies. For example, incorporating multiple phenotypes (outcomes) into a joint model with high-dimensional mediators can improve the efficiency, e.g., the joint model of survival and longitudinal [47], survival and recurrent events [48,49]. Another example is to incorporate multiple exposures into high-dimensional mediation analysis with survival outcome, since both lung cancer and methylation are associated with many risk factors. Besides, with high dimensionality and mediation model, adding interaction terms increase the model complexity dramatically. Since the selection and estimation of interaction terms are of much different interpretation, we consider this to be beyond the scope of the current paper. However, it will be interesting to consider a further study of high dimensional mediator selection with interaction terms. Further researches are needed for these method developments.

## Materials and methods

### Notations and high-dimensional mediation models

**Fig 1:**
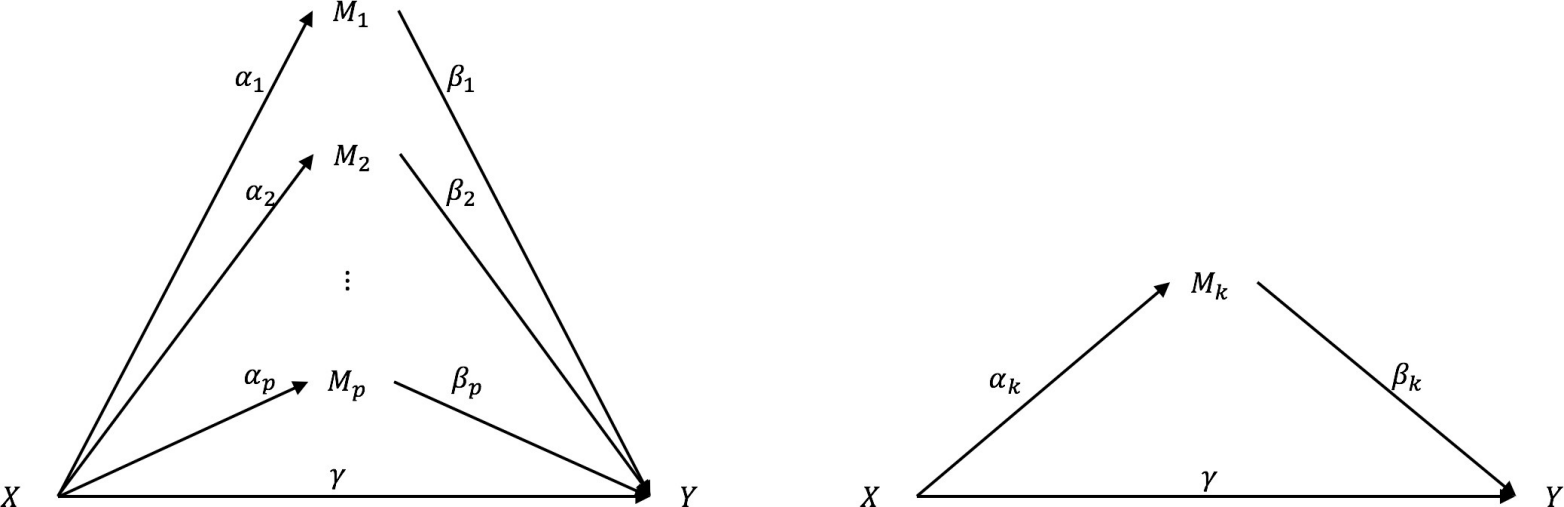
**(Left)** The directed acyclic graph describes high-dimensional mediation with the *p* mediators assumed to be uncorrelated with one another. **(Right)** The relationship of the three-variable path diagram used to represent standard mediation framework.

Let *D*_*i*_ denote the time from onset to an event (death) and *C*_*i*_ be the potential censoring time. The observed survival time is *T*_*i*_ = min(*D*_*i*_,*C*_*i*_), and the failure indicator can be expressed as *δ*_*i*_ = *I*(*D*_*i*_≤*C*_*i*_), where *I*(∙)is an indicator function. Let *X*_*i*_ be the exposure (smoking status, i.e., smoker or non-smoker), *Z*_*i*_ be the other *q* baseline covariates, and *M*_*i*_ = (*M*_1*i*_,*M*_2*i*_,⋯,*M*_*pi*_)^*T*^ be a p-dimensional mediator vector (contains all the methylation information) for individual *i*,*i* = 1,2,⋯,*n*, and *p*≫*n*. Fig 1 illustrates the relationship among exposure (*X*), mediators (*M*_*k*_), and time-to-event outcome (*Y*).

Mediation models are used to model the mechanism of the exposure’s effect on the outcome mediated by the mediators. In the context of time-to-event data, the rate at time *t* means the probability of experiencing death within the next unit of time, given that a patient is still alive right before time *t*. Cox proportional hazards model [50] uses the hazard ratio as an expression of how many times greater the rate is for the smoking group relative to the non-smoking group. For the survival outcome, we consider the following regression models to assess the mediation effects with high-dimensional mediators:

$$\lambda_{i}(t|X_{i},M_{i})=\lambda_{0}(t)\exp\left\{\gamma X_{i}+\theta^{T}Z_{i}+\beta_{1}M_{1i}+\cdots \beta_{p}M_{pi}\right\},$$ (1)

$$M_{ki}=c_{k}+\alpha_{k}X_{i}+\vartheta^{T}Z_{i}+e_{ki},k=1,2,\cdots ,p,$$ (2)

where Eq (1) is the Cox proportional hazards model which describes the relationship between the exposure *X*, mediators *M* and the time-to-event variable; Eq (2) characterizes how the exposure variables influence the mediators; *λ*_0_(*t*) is the baseline hazard function; *Z* is the baseline covariates including gender, age and other baseline characteristics; γ is the direct effect of the exposure on the outcome; *β* = (*β*_1_,⋯,*β*_*p*_)^*T*^ is the parameter vector relating the mediators to the outcome adjusting for the effect of exposure; *α* = (*α*_1_,⋯,*α*_*p*_)^*T*^ is the parameter vector relating the exposure to the mediators; *c*_*k*_ is the intercept term and *e*_*ik*_~*N*(0,*σ*^2^) is the residual.

### Assumptions

Assumptions about absence of confounders should be made if one intends to obtain causal conclusion from an analysis. Here, *T*(*x*,*m*_1_,⋯,*m*_*p*_) denotes the survival time when the exposure be set to *x* and the mediator is set to *m*_*k*_,*k* = 1,2,⋯,*p*, and *M*_*k*_(x*) denotes the value of the mediator when the exposure is set to x*. *Z* denotes baseline covariates such as age and gender. Except for the assumption of consistency[51], based on Huang and Yang (2017) [19], we also assume the following hypothesis which is of great importance in subsequent derivations.

(A1). *X*⊥*T*(*x*,*m*_1_,⋯,*m*_*p*_)|*Z*; that is no unmeasured confounders between the exposure and outcome.
(A2). For any *k* = 1,2,⋯,*p*, *M*_*k*_⊥*T*(*x*,*m*_1_,⋯,*m*_*p*_)|*X*,*Z*; that is no unmeasured confounders between the mediators and outcome.
(A3). For any *k* = 1,2,⋯,*p*, *X*⊥*M*_*k*_|*Z*; that is no unmeasured confounders between the exposure and mediator.
(A4). For any *k* = 1,2,⋯,*p*, *M*_*k*_(x*)⊥*T*(*x*,*m*_1_,⋯,*m*_*p*_)|*Z*; that is no measured or unmeasured exposure-dependent confounders between the mediators and outcome, where x* is the intervention for the exposure *X* with different value than *x*.

### Proposed procedure

**Fig 2:**
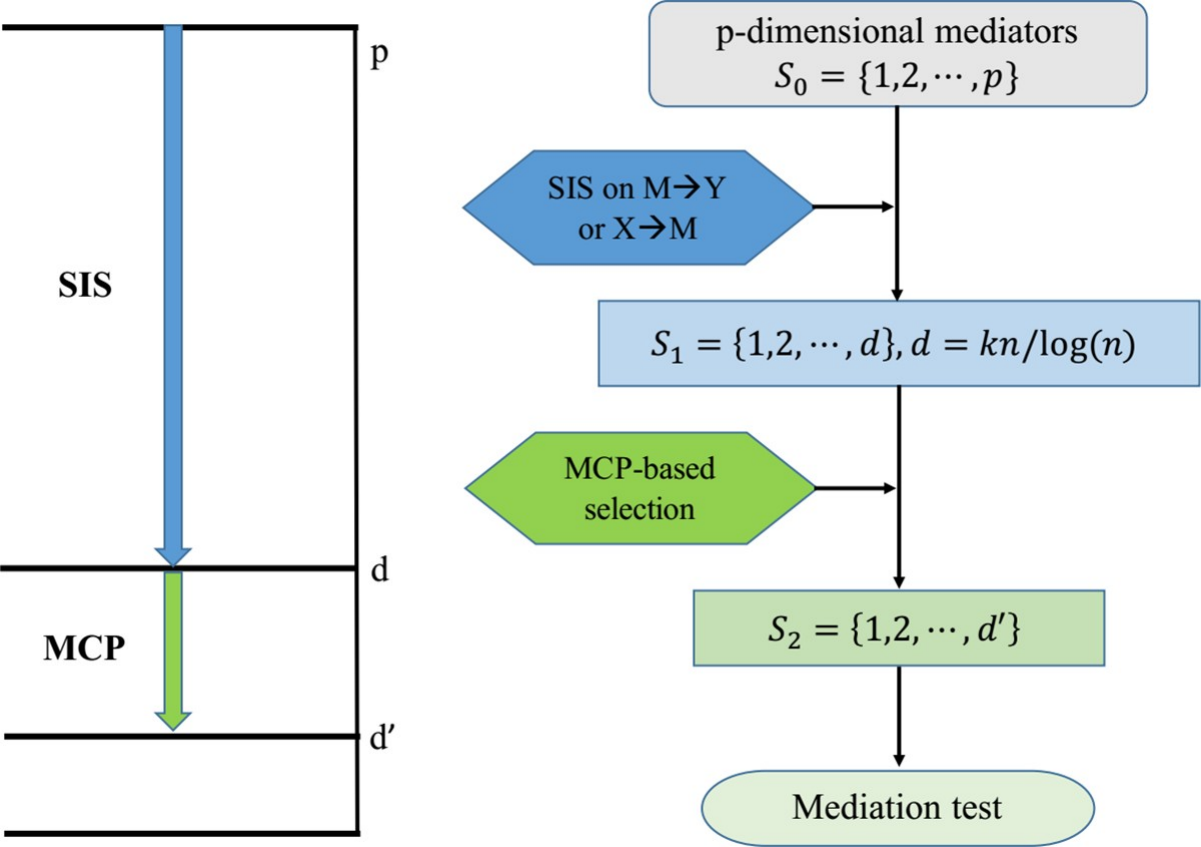
Overall workflow for high-dimensional mediation analysis. The workflow includes the main processes: (a) using SIS technique for preliminary screening; (b) conducting MCP-based variable selection; (c) testing for mediation effects.

For estimation in the survival component, the corresponding log-partial likelihood function of (1) is given by

$$l_{n}(\beta )=\sum_{i=1}^{n}\delta_{i}\{P_{i}^{T}Q-\log\left[\sum_{l\in R_{i}}\exp\left(P_{l}^{T}Q\right)\right]\},$$ (3)

where *R*_*i*_ = {*l*:*T*_*l*_≥*T*_*i*_} is the at-risk set; *P*_*i*_ = (*X*_*i*_,*Z*_*i*_,*M*_1*i*_,⋯,*M*_*pi*_)^*T*^ and *Q* = (*γ*,*θ*,*β*_1_,⋯,*β*_*p*_)^T^. The goal of variable selection is to identify $S=\{k:\;{\hat{\beta }}_{k}\neq 0\}$, a subset of Q, which contains all the variables that are the significant mediators between the exposure and the outcome. Nevertheless, the number of mediators *p* is much larger than the sample size *n*, and the traditional statistics methods for Cox regression analysis fail to work in (3). To deal with this problem, we will first apply sure independence screening (SIS) [37] method to identify a subset *S*_1_ = {*k*:1≤*k*≤*p*} of size *d* = [*kn*/*log*(*n*)] which are the mediators with strong correlation value for the response variable. We will then conduct variable selection via MCP-based Cox model within the subset *S*_1_. Finally, we estimate the direct and indirect effects and perform significance test. Fig 2 illustrates the overall workflow for high-dimensional mediation analysis. Details of the proposed procedure are in the following steps:

**Step 1.** (*Preliminary screening*) Based on SIS [37], for *k* = 1,⋯,*p*, we select a subset *S*_1_ = {*k*: 1≤*k*≤p} of size *d* = [2*n*/*log*(*n*)]. For the mediators in *S*_1_ are among the top *d* strong correlation value for the response variable.
  Sure independence screening, which is based on correlation learning, has been a general technique to reduce dimensionality from high to a small scale that is below the sample size. Here we use *d* = 2*n*/*log*(*n*) in the place of *d* = *n*/*log*(*n*) to increase the probability for identifying important mediators [37], considering that both *α*_*k*_ and *β*_*k*_ have to be selected as nonzero to ensure a specific mediator to be selected.
**Step 2.** (*MCP-penalized variable selection*) Among all the screened mediators *M*_*k*_∈*S*_1_ from the Step 1, we further identify the subset $S_{2}=\{k:\;{\hat{\beta }}_{k}\neq 0\}$ via the penalized log-partial likelihood optimization
  $$\hat{\beta }={argmax}_{\beta }\{l_{n}(\beta )-\sum_{k=1}^{p}P_{\lambda }(\beta_{k})\},$$ (4)
  where *l*_*n*_(*β*) is showed in Eq (3); *P*_*λ*_(∙) is the penalty function that depends on the regularization parameter *λ*>0, which controls the strength of regularization. Tibshirani (1997) [52] proposed a penalized reweighted least squares method to solve (4). The detailed calculation and derivation process of the above equation is provided in the Supporting Information (S2 Text).
  Here, we adopt the minimax concave penalty (MCP) proposed by Zhang (2010) [38] with the following derivative function
  $$P_{\lambda }^{\prime }(\beta_{k})=\frac{{\left(a\lambda -{|\beta }_{k}|\right)}_{+}}{a\lambda },$$
  where *a*>1 is a shape parameter. Breheny and Huang (2011) implemented the MCP procedure with the R package *ncvreg* [53]. Here we prefer MCP approach over other penalty, e.g. lasso, as MCP is a fast, nearly unbiased and accurate approach of penalized variable selection in high-dimensional context. Besides, it has the oracle property which can select the correct model with probability tending to 1.
**Step 3.** (*Effect decomposition*) Lange and Hansen (2011) have studied direct and indirect effects for single mediator in a survival context with Aalen additive hazards model [15]. The idea is to use the counterfactual rate difference as the effect measure of the exposure changing from *x* to x*. Huang and Yang (2017) extend to two mediators with Cox model [19]. To extend the decomposition of direct and indirect effect to high-dimensional mediators model, we first approximate the counterfactual outcome defined as log hazard as follows
  $$\begin{array}{l} log\lambda (T(x,M_{1}^{x^{*}},\cdots ,M_{p}^{x^{*}});t|Z)\\ =log\lambda_{0}(t)+\theta^{T}Z+\frac{1}{2}\sigma_{W_{\beta }}^{2}+\beta_{1}(c_{1}+\vartheta^{T}Z)\cdots +\beta_{p}(c_{p}+\vartheta^{T}Z)\\ +\gamma x+(\alpha_{p}\beta_{p}+\cdots +\alpha_{p}\beta_{p})x^{*} \end{array}$$
  where $\sigma_{W_{\beta }}^{2}=\sum \beta_{k}^{2}\sigma_{M_{k}}^{2}$. Derivation of the above expression is provided in the Supporting Information (S3 Text). Then, we can express the direct effect and total indirect effect on log hazard ratio by using the above expression as
  $$\begin{array}{l} \Delta_{X\to Y}=log\lambda (T(x^{*},M_{1}(x),\cdots ,M_{p}(x));t|Z)-log\lambda (T(x,M_{1}(x),\cdots ,M_{p}(x));t|Z)\\ =(x^{*}-x)\gamma , \end{array}$$
  $$\begin{array}{l} \Delta_{X\to M\to Y}=log\lambda (T(x^{*},M_{1}(x^{*}),\cdots ,M_{p}(x^{*}));t|Z)-log\lambda (T(x^{*},M_{1}(x),\cdots ,M_{p}(x));t|Z)\\ =(x^{*}-x)(\alpha_{1}\beta_{1}+\cdots +\alpha_{p}\beta_{p}), \end{array}$$
  and the total effect is the sum of the direct and total indirect effects.
**Step 4.** (*Significance test*) For *k*∈*S*_2_, a variable *M*_*k*_ is considered as a mediator between the exposure and outcome only if the indirect effect is significant. Here, we consider two methods to test the mediation effects, including Sobel test (i.e., product method [54]) and joint significant test (i.e., causal steps method [55]). Followed with the Sobel test for indirect effect, we have the p-value for testing the null hypothesis *H*_0_: *α*_*k*_*β*_*k*_ = 0 of no indirect effect
  $$P_{raw,k}=2\{1-\phi (\frac{\left|{\hat{\alpha }}_{k}{\hat{\beta }}_{k}\right|}{{\hat{\sigma }}_{\alpha_{k}\beta_{k}}})\},$$
  where ${\hat{\sigma }}_{\alpha_{k}\beta_{k}}$ is the estimate of the Sobel standard error (SE) [54]. We have the revised p-value via the Bonferroni’s method in order to adjust for multiple comparisons
  $$P_{k}=\min\left\{P_{raw,k}∙|S_{2}|,1\right\},$$ (5)
  where |*S*_2_| is the number of elements in set *S*_2_. The joint significant test for indirect effect is based on the path-specific (i.e., *X*→*M* and *M*→*Y*) *P*-values [55] and does not provide an estimate. Hence, we can reject the null hypothesis of no *IE*_*k*_ if *P*_*k*_<0.05, and conclude that the variable *M*_*k*_ is the significant mediator between the exposure and outcome.

## Supporting information

S1 Text SIS on path X→M. (DOC)

S2 Text The detailed calculation and derivation process of the MCP-penalized variable selection. (DOC)

S3 Text The deviation for the effect decomposition. (DOC)

S1 Table Comparison of mediator selection between proposed method and one-step method (p = 10000, 500 replicates). (XLSX)

S2 Table Comparison of mediator selection between proposed method and naive method (p = 10000, 500 replicates). (XLSX)

S3 Table Comparison of mediator selection between MCP-based method and LASSO-based method (p = 10000, 500 replicates). (XLSX)

S4 Table Accuracy of mediator selection with different number of significant mediators (p = 10000, 500 replicates). (XLSX)

S5 Table Accuracy of mediator selection with higher censoring rate (p = 10000, 500 replicates). (XLSX)

S6 TableAccuracy of mediator selection with dependence among mediators (p = 10000, Cen = 15%, 500 replicates, ${\tilde{M}}_{k}=M_{k}+\sum_{l=1}^{k-1}\delta_{lk}M_{l}$).(XLSX)

S7 Table Estimation for *α*_*k*_*β*_*k*_ with the increase of noise (n = 750, p = 10000, 100 replicates). (XLSX)

S8 Table Estimation for α_k_β_k_ with the increase of dimension of mediators (n = 750, 100 replicates). (XLSX)
