# Supplementary material for: High-dimensional mediation analysis in survival models
Source: PLoS Comput Biol. 2020 Apr 17;16(4):e1007768. doi: 10.1371/journal.pcbi.1007768 (PMC7190184; doi:10.1371/journal.pcbi.1007768)
Supplement: S1 Text — (DOC) [file pcbi.1007768.s001.doc]

**S1 Text. SIS on path X🡪M**

For $k=1, \cdots, p$, we select a subset $S_{1}=\left\{ k:1\leq k\leq p \right\}$ of size $d$. For the mediators in $S_{1}$ are among the top $d$ largest effect for the regression model $M_{k}\sim X+Z$ which is similar with the correlation between mediator and exposure.
