# Supplementary material for: High-dimensional mediation analysis in survival models
Source: PLoS Comput Biol. 2020 Apr 17;16(4):e1007768. doi: 10.1371/journal.pcbi.1007768 (PMC7190184; doi:10.1371/journal.pcbi.1007768)
Supplement: S2 Text — (DOC) [file pcbi.1007768.s002.doc]

**S2 Text. The detailed calculation and derivation process of the MCP-penalized variable selection.**

Among all the screened mediators $M_{k}\in S_{1}$ from the Step 1 we further identify the subset $S_{2}=\left\{ k: \hat{\beta}_{k}\neq0 \right\}$ via the penalized log-partial likelihood optimization

$\hat{\text{β}}\text{=}\mathrm{argmax}_{\beta}\left\{ l_{n}(\beta)-\sum_{k=1}^{p} P_{\lambda}\left( \beta_{k} \right) \right\}$, (4)

where $l_{n}(\beta)$ is showed in equation (3); $P_{\lambda}(\cdot)$ is the penalty function that depends on the regularization parameter $\lambda>0$, which controls the strength of regularization. Tibshirani (1997) [1] proposed a penalized reweighted least squares method to solve (4).

Let $P$ be the $n\times(p+q+1)$ design matrix and define $\eta=P^{T}Q$. Let $l_{n}^{'}(\eta_{i})$ and $l_{n}^{''}(\eta_{i})$ denote the gradient vector and Hessian matrix of the log-partial likelihood $l_{n}(\beta)$ with respect to $\eta_{i}$ (the *i*th component of $\eta$, $i=1,\cdots,n$) respectively as following:

$$l_{n}^{'}\left( \eta_{i} \right)=\frac{\partial}{\partial\eta_{i}}l_{n}\left( \eta\right)=\delta_{i}-e^{\eta_{i}}\sum_{l=1}^{n} \frac{\delta_{l}I\left( T_{i}\geq T_{l} \right)}{\sum_{j=1}^{n} I\left( T_{j}\geq T_{l} \right)e^{\eta_{j}}},$$

$l_{n}^{''}\left( \eta_{i} \right)=\frac{\partial^{2}}{\partial\eta_{i}^{2}}l_{n}\left( \eta\right)=-e^{\eta_{i}}\sum_{l=1}^{n} \frac{\delta_{l}I\left( T_{i}\geq T_{l} \right)}{\sum_{j=1}^{n} I\left( T_{j}\geq T_{l} \right)e^{\eta_{i}}}+e^{2\eta_{i}}\sum_{l=1}^{n} \frac{\delta_{l}I\left( T_{i}\geq T_{l} \right)}{{(\sum_{j=1}^{n} I\left( T_{j}\geq T_{l} \right)e^{\eta_{j}})}^{2}}$.

A second-ordered Taylor series expansion of the log-partial likelihood centered at $\hat{\beta}$ leads to

$l_{n}\left( \beta\right)\approx\frac{1}{2}\left[ z\left( \hat{\eta} \right)-P^{T}Q \right]^{T}l_{n}^{''}(\hat{\eta})\left[ z\left( \hat{\eta} \right)-P^{T}Q \right]$*,*

where $\hat{\eta}=P^{T}\hat{Q}$ and $z\left( \hat{\eta} \right)=\hat{\eta}-l_{n}^{''}\left( \hat{\eta} \right)^{-1}l_{n}^{'}(\hat{\eta})$. According to Hastie and Tibshirani (1990), the matrix $l_{n}^{''}(\hat{\eta})$ is non-diagonal which may increase the computation complexity [2]. In order to speed up the algorithm, Tibshirani (1997) argued to approximate the matrix with a diagonal one (set off-diagonal elements as zero) [1]. Let $\omega{(\hat{\eta})}_{i}$ denote the $i\mathrm{th}$ diagonal entry of $l_{n}^{''}(\hat{\eta})$ for subject $i$, following [1], we compute $S_{2}=\left\{ k: \hat{\beta}_{k}\neq0 \right\}$ by minimizing the MCP-penalized reweighted least squares

$\frac{1}{n}\sum_{i=1}^{n} \omega\left( \hat{\eta} \right)_{i}{[z\left( \hat{\eta} \right)_{i}-P_{i}^{T}Q]}^{2}+\sum_{k=1}^{p} P_{\lambda}(\beta_{k})$*.*

We adopt the minimax concave penalty (MCP) proposed by Zhang (2010) [3] with the following derivative function

$P_{\lambda}^{'}\left( \beta_{k} \right)=\frac{{(a\lambda-{|\beta}_{k}|)}_{+}}{a\lambda}$*,*

where $a>1$ is a shape parameter.

**Reference**

1. Tibshirani R. The lasso method for variable selection in the cox model. Statistics in Medicine. 1997;16(4):385-95.

2. Hastie T, Tibshirani R. Generalized Additive Models. Chapman and Hall/CRC1990.

3. Zhang CH. Nearly unbiased variable selection under minimax concave penalty. Annals of Statistics. 2010;38(2):894-942.
