# Supplementary material for: High-dimensional mediation analysis in survival models
Source: PLoS Comput Biol. 2020 Apr 17;16(4):e1007768. doi: 10.1371/journal.pcbi.1007768 (PMC7190184; doi:10.1371/journal.pcbi.1007768)
Supplement: S3 Text — (DOC) [file pcbi.1007768.s003.doc]

**S3 Text. Effect Decomposition**

The high-dimensional mediation models can be expressed as:

$\lambda_{i}\left( t | X_{i},M_{i} \right)=\lambda_{0}\left( t \right)\exp\left\{ \gamma X_{i}+\theta^{T}Z_{i}+\beta_{1}M_{1i}+\cdots\beta_{p}M_{pi} \right\}$, (1)

$M_{ki}=c_{k}+\alpha_{k}X_{i}+\vartheta^{T}Z_{i}+e_{ki}, k=1,2,\cdots,p$*,* (2)

Assumptions:

(A1). $X\perp T(x,m_{1},\cdots,m_{p})|Z$; that is no confounding between the exposure and outcome.

(A2). For any $k=1,2,\cdots,p$, $M_{k}\perp T(x,m_{1},\cdots,m_{p})|X,Z$; that is no confounding between the mediators and outcome, conditional on the exposure.

(A3). For any$k=1,2,\cdots,p$, $X\perp M_{k}|Z$; that is no confounding between the exposure and mediator.

(A4). For any $k=1,2,\cdots,p$, $M_{k}(x^{*})\perp T(x,m_{1},\cdots,m_{p})|Z$; that is no exposure-dependent confounders between the mediators and outcome, where $x^{*}$ is the intervention for the exposure $X$ with different value than $x$.

Lange and Hansen (2011) have studied direct and indirect effects for single mediator in a survival context with Aalen additive hazards model [1]. The idea is to use the counterfactual rate difference as the effect measure of the exposure changing from $x$ to $x^{*}$. Huang and Yang (2017) present the decomposition of the total effect with two mediators Huang and Yang (2). Here, we extend to the high-dimensional mediators using Cox model.

Except for the assumption of consistency [3], based on the above assumptions. The cumulative distribution function of the counterfactual survival time can be expressed as

$$F_{T\left( x,M_{1}(x^{*}),\cdots,M_{p}(x^{*}) \right)}\left( t | Z \right)=\int\cdots\int F_{T\left( x,m_{1},\cdots,m_{2} \right)}\left( t | Z,M_{1}\left( x^{*} \right)=m_{1},\cdots,M_{p}\left( x^{*} \right)=m_{p} \right)dF_{M_{1}}\left( m_{1} | Z,m_{1} \right)\cdots dF_{M_{p}}\left( m_{p} | Z,m_{p} \right)=\int\cdots\int F_{T\left( x,m_{1},\cdots,m_{2} \right)}\left( t | Z \right)dF_{M_{1}}\left( m_{1} | Z \right)\cdots dF_{M_{p}}\left( m_{p} | Z \right) \left( A4 \right) =\int\cdots\int F_{T\left( x,m_{1},\cdots,m_{2} \right)}\left( t | Z \right)dF_{M_{1}}\left( m_{1} | Z,x^{*} \right)\cdots dF_{M_{p}}\left( m_{p} | Z,x^{*} \right) \left( A3 \right)=\int\cdots\int F_{T\left( x,m_{1},\cdots,m_{2} \right)}\left( t | Z,x \right)dF_{M_{1}}\left( m_{1} | Z,x^{*} \right)\cdots dF_{M_{p}}\left( m_{p} | Z,x^{*} \right) \left( A1 \right)=\int\cdots\int F_{T\left( x,m_{1},\cdots,m_{2} \right)}\left( t | Z,x,m_{1},\cdots,m_{p} \right)dF_{M_{1}}\left( m_{1} | Z,x^{*} \right)\cdots dF_{M_{p}}\left( m_{p} | Z,x^{*} \right). (A2)$$

Hence, the probability density function has the form as

$$f_{T\left( x,m_{1},\cdots,m_{2} \right)}\left( t | Z \right)=dF_{T\left( x,m_{1},\cdots,m_{2} \right)}\left( t | Z \right)=\int\cdots\int dF_{T\left( x,m_{1},\cdots,m_{2} \right)}\left( t | Z,x,m_{1},\cdots,m_{p} \right)dF_{M_{1}}\left( m_{1} | Z,x^{*} \right)\cdots dF_{M_{p}}\left( m_{p} | Z,x^{*} \right)=\int\cdots\int f_{T\left( x,m_{1},\cdots,m_{2} \right)}\left( t | Z,x,m_{1},\cdots,m_{p} \right)dF_{M_{1}}\left( m_{1} | Z,x^{*} \right)\cdots dF_{M_{p}}\left( m_{p} | Z,x^{*} \right)$$

Based on (1), the log-transformed Cox proportional hazards model can be expressed as:

$\log\lambda_{i}\left( t \right)=\log\lambda_{0}\left( t \right)+\gamma X_{i}+\theta^{T}Z_{i}+W_{\beta i}$,

where $W_{\beta i}=\beta_{1}M_{1i}+\beta_{2}M_{2i}+\cdots+\beta_{p}M_{pi}$. $W_{\beta i}$ is a function of $x^{*}$, following a normal distribution $G_{W_{\beta}}: W_{\beta}(x^{*})\sim N(\mu_{W_{\beta}},\sigma_{W_{\beta}}^{2})$, where $\mu_{W_{\beta}}=\beta_{1}\left( c_{1}+\vartheta^{T}Z+\alpha_{1}x^{*} \right)+\cdots+\beta_{p}\left( c_{p}+\vartheta^{T}Z+\alpha_{p}x^{*} \right)$ and $\sigma_{W_{\beta}}^{2}=\beta_{1}^{2}\sigma_{M_{1}}^{2}+\cdots+\beta_{p}^{2}\sigma_{M_{p}}^{2}$.

According to the assumptions and the results derived in the above, the counterfactual outcome defined as log hazard can be expressed as follows:

$$\log\lambda\left( T\left( x,M_{1}\left( x^{*} \right),\cdots,M_{p}\left( x^{*} \right) \right);t | Z \right)=log\frac{\int f_{T\left( x,M_{1}\left( x^{*} \right),\cdots,M_{p}\left( x^{*} \right) \right)}(t|Z)}{1-\int F_{T\left( x,M_{1}\left( x^{*} \right),\cdots,M_{p}\left( x^{*} \right) \right)}(t|Z)}=\log\frac{\int f_{T}(t|x,Z)dG_{W_{\beta}}(x^{*})}{1-\int F_{T}(t|x,Z)dG_{W_{\beta}}(x^{*})}=\log\frac{\int\lambda(t|x,Z)e^{-\lambda(t|x,Z)}dG_{W_{\beta}}(x^{*})}{\int e^{-\lambda(t|x,Z)}dG_{W_{\beta}}(x^{*})}\approx\log\int\lambda\left( t | x,Z \right)dG_{W_{\beta}}\left( x^{*} \right).$$

The last equation is an approximation by assuming the outcome is rare and then $e^{-\lambda\left( t | x,Z \right)}\approx1$. Hence,

$$\log\lambda\left( T\left( x,M_{1}\left( x^{*} \right),\cdots,M_{p}\left( x^{*} \right) \right);t | Z \right)\approx log\int\lambda\left( t | x,Z \right)dG_{W_{\beta}}\left( x^{*} \right)=\log\lambda_{0}\left( t \right)+\theta^{T}Z+\gamma x+\mu_{W_{\beta}}+\frac{1}{2}\sigma_{W_{\beta}}^{2}=log\lambda_{0}\left( t \right)+\theta^{T}Z+\frac{1}{2}\sigma_{W_{\beta}}^{2}+\beta_{1}\left( c_{1}+\vartheta^{T}Z \right)\cdots+\beta_{p}(c_{p}+\vartheta^{T}Z)+\gamma x+(\alpha_{p}\beta_{p}+\cdots+\alpha_{p}\beta_{p})x^{*},$$

and the second equation utilizes the property of moment generating function of normal random variable since $W_{\beta}$ is normally distributed.

Through extending the decomposition of direct and indirect effect to high-dimensional mediators model, we can express the total causal effect on log hazard ratio as

$$\log\lambda\left( T\left( x^{*},M_{1}\left( x^{*} \right),\cdots,M_{p}\left( x^{*} \right) \right);t|Z \right)-\log\lambda\left( T\left( x,M_{1}\left( x \right),\cdots,M_{p}\left( x \right) \right);t|Z \right) = \log\lambda\left( T\left( x^{*},M_{1}\left( x^{*} \right),\cdots,M_{p}\left( x^{*} \right) \right);t|Z \right)-\log\lambda\left( T\left( x^{*},M_{1}\left( x \right),\cdots,M_{p}\left( x \right) \right);t|Z \right)+log\lambda\left( T\left( x^{*},M_{1}\left( x \right),\cdots,M_{p}\left( x \right) \right);t|Z \right)-\log\lambda\left( T\left( x,M_{1}\left( x \right),\cdots,M_{p}\left( x \right) \right);t|Z \right)=\left( x^{*}-x \right)\left( \alpha_{1}\beta_{1}+\cdots+\alpha_{p}\beta_{p} \right)+\left( x^{*}-x \right)\gamma=log(IE)+log(DE),$$

where TE denote the total effect; DE denote the direct effect; and IE denote the indirect effect. We can also get the path-specific effects (${IE}_{k}$) for each mediator $M_{k}$.

**Reference**

1. Lange T, Hansen JV. Direct and indirect effects in a survival context. Epidemiology. 2011;22(4):575.

2. Huang YT, Yang HI. Causal mediation analysis of survival outcome with multiple mediators. Epidemiology. 2017;28(3):370.

3. VanderWeele TJ. Marginal structural models for the estimation of direct and indirect effects. Epidemiology. 2009;20(1):18-26.
